# Rapid Capturing and Chemiluminescent Sensing of Programmed Death Ligand-1 Expressing Extracellular Vesicles

**DOI:** 10.3390/bios12050281

**Published:** 2022-04-28

**Authors:** Adeel Khan, Kaili Di, Haroon Khan, Nongyue He, Zhiyang Li

**Affiliations:** 1 State Key Laboratory of Bioelectronics, School of Biological Science and Medical Engineering, National Demonstration Center for Experimental Biomedical Engineering Education, Southeast University, Nanjing 210096, China; adeel9925@seu.edu.cn; 2 Department of Clinical Laboratory Medicine, the Affiliated Drum Tower Hospital, Medical School of Nanjing University, Nanjing 210008, China; mg1835040@smail.nju.edu.cn (K.D.); lizhiyang@nju.edu.cn (Z.L.); 3 Neuroscience and Neuroengineering Research Center, Med-X Research Institute, School of Biomedical Engineering, Shanghai Jiao Tong University, Shanghai 200240, China; harry_884489@sjtu.edu.cn

**Keywords:** extracellular vesicles, Fe_3_O_4_@TiO_2_, PD-L1, aptasensor, chemiluminescence, lung cancer, non-invasive diagnosis

## Abstract

Cancer specific extracellular vesicles (EVs) are of significant clinical relevance, for instance programmed death ligand-1 (PD-L1) expressing EVs (PD-L1@EVs) have been shown to be ideal biomarker for non-invasive diagnosis of cancer and can predate the response of cancer patients to anti-PD-1/PD-L-1 immunotherapy. The development of sensitive and straightforward methods for detecting PD-L1@EVs can be a vital tool for non-invasive diagnosis of cancer. Most of the contemporary methods for EVs detection have limitations such as involvement of long and EV’s loss prone isolation methods prior to detection or they have employed expensive antibodies and instruments to accomplish detection. Therefore, we designed an ultracentrifugation-free and antibody-free sensing assay for PD-L1@EV by integrating Titanium oxide (TiO${}_{2}$) coated magnetic beads (Fe${}_{3}$O${}_{4}$@TiO${}_{2}$) rapid capturing of EVs from undiluted serum with aptamers specificity and chemiluminescence (CL) sensitivity. To accomplish this we used Fe${}_{3}$O${}_{4}$@TiO${}_{2}$ beads to rapidly capture EVs from the undiluted patient serum and added biotin labelled PD-L1 aptamer to specifically recognize PD-L1@EVs. Later, added streptavidin-modified Alkaline phosphates (ALP) taking advantage of biotin-streptavidin strong binding. Addition of CDP-star, a chemiluminescent substrate of ALP, initiates the chemiluminiscense that was recorded using spectrophotometer. The sensing assay showed high sensitivity with limit of detection (LOD) as low as $2.584\times 10^{5}$ EVs/mL and a wider linear correlation of CL intensity (a.u.) with the concentration of PD-L1@EVs from $10^{5}$ to $10^{8}$ EVs/mL. To examine the clinical utility of sensing assay we used undiluted serum samples from lung cancer patients and healthy individuals and successfully discern between healthy individuals and lung cancer patients. We are optimistic that the sensing assay can ameliorate our ability to be able to diagnose lung cancer non-invasively and can be helpful to predate the patient’s response to anti-PD-1/PD-L1 immunotherapy.

## 1. Introduction

Extracellular vesicles (EVs) are membrane enclosed nano-size-entities (50–1000 nm diameter) with a biologically active cargo. A plethora of cell types release EVs. The presence of EVs has been reported in majority of body fluids for instance blood, urine, saliva and milk, hence, can be amassed non-invasively [1]. Cancer cells release significantly more EVs than normal cells. A growing body of evidence propose that EVs released by cancer cells can be diagnostic markers for a various cancers, valuable for cancer monitoring, early detection of cancer relapse, and even response to an anti-cancer therapy [2]. One such example are the EVs expressing Programmed death ligand-1 (PD-L1). PD-L1 also known as CD274 is a crucial immune check point protein that has been reported to be expressed on the surface of cancer cells as well as cancer cells derived EVs [3]. EVs with PD-L1 marker (PD-L1@EVs) can cause strong immunosuppressive effects [4]. To accomplish immunosuppression the PD-L1@EVs binds the activated T cells via PD-1 receptor resulting in cessation of their proliferation, cytokine production and cytotoxicity. PD-L1@EVs can be diagnostic markers and can also stratify between patients responding and non-responding to anti-PD-1/PD-L1 immunotherapy [3,5].

Lung cancer (LC) is one of the deadliest cancers both in males and females with a dismal 5-years survival rate. The new cases of LC are expanding with alarming rate [6]. Mostly LC patients are diagnosed at late stages, when the treatment options are narrow. It is therefore crucial to develop techniques that aid in the non-invasive early diagnosis of LC; a stage more amenable to treatment. EVs’ abundance in body fluids and non-invasive collection confer EVs a status of ideal biomarker for diagnosis of LC [7]. To develop excellent detection method for EVs an important step is efficient and rapid capturing of EVs from clinical samples. EVs amassment by ultracentrifugation/commercial kits is a laborious and long operation (from several hours to overnight). Ultrafiltration based isolation of EVs is simple and fast but high loss of EVs limits its utility. Polymer based EVs precipitation is quite simple but low specificity, and co-enrichment of contaminates with EVs discourage its use [8]. These canonical limitations of traditional methods intensify the need for better enrichment strategies [9]. Advance methods such as acoustofluidic methods yield EVs comparable to ultracentrifugation by using a very small sample volume, however research is still needed to optimize the combined acoustic/microfluidic forces for efficient control of nanoparticles like EVs that are very small in size and buoyant [10]. In the recent past advances in magnetic nanopartickes/beads based EVs enrichment and detection platforms have shown remarkable progress and are favoured for their simplicity, robustness and cost effectiveness [11]. Recently, Gao and fellows developed magnetic beads with titanium oxide coating (Fe${}_{3}$O${}_{4}$@TiO${}_{2}$) for the enrichment of exosomes from serum. The enrichment of exosomes with Fe${}_{3}$O${}_{4}$@TiO${}_{2}$ is assisted by strong bidentate binding between titanium oxide and phosphate group of the lipid bilayer of exosomes. In the complex biological systems for instance serum, the hydrophilic phosphate heads of phospholipids tend to be exposed on outer side of lipid bilayer. Fe${}_{3}$O${}_{4}$@TiO${}_{2}$ based selective enrichment of exosomes is fast, efficient, and free of contamination due to non-specific adsorption [12]. The use of Fe${}_{3}$O${}_{4}$@TiO${}_{2}$ beads greatly reduced the EVs’ isolation time and reduced the loss of EVs during the enrichment step without affecting its proteome [13].

By targeting the surface markers of EVs many strategies have been developed for EVs detection such as colorimetric [14], fluorescence [15] and electrochemical [16]. The detection of EVs with tumor specific markers (such as PD-L1) is an interesting and worth exploring avenue of research in oncology [17]. Some methods reporting the detection of PD-L1@EVs have achieved good sensitivity, but are either based on sophisticated instruments such as flow cytometry [18] and droplet digital PCR [17] or are time consuming [19]. Many methods reporting the detection of PD-L1@EVs had used antibodies for PD-L1 recognition. However, antibodies have canonical limitations such as batch to batch variations, poor stability and high cost [14]. An alternative to antibodies is aptamer that can exert antibodies-mimic function. Aptamers are small size short oligonucleotides that can be synthesized synthetically to exhibit high specific binding affinity. They are stable, less pricey, amenable to chemical modifications and can be synthesized in large scale with controllable batch to batch variations, Therefore, aptamers are lucrative option for development of biosensing tools that can be applied in the physiological environment. Recently, CD63 specific aptamers [20] and PD-L1 specific aptamers [17] based detection platforms for EVs have shown excellent sensitivity and specificity.

As EVs are considered ideal biomarker for the diagnosis of variety of diseases, research on development of new detection platforms for EVs is rapidly expanding [21]. An important improvement in EVs detection methods is to enrich EVs directly from the serum without employing traditional EVs enrichment steps such as ultracentrifugation/ultrafiltration/expensive commercial kits or antibody coated magnetic beads [22]. We therefore, designed a chemiluminescent sensing assay for the detection of PD-L1@EVs by integrating Fe${}_{3}$O${}_{4}$@TiO${}_{2}$ for rapid enrichment of EVs from a small volume of undiluted serum with PD-L1 aptamers specificity [23], and chemiluminescence sensitivity. Chemiluminescence is light emission due to chemical reaction, chemiluminescence based detection platforms have the potential to achieve lower detection limits and wide linear calibration range [24], therefore, can be valuable for clinical diagnosis [25] such as clinical diagnosis of cancer markers and EVs [26].

## 2. Materials and Methods

### 2.1. Aptamers Sequences and Auxiliary Reagents

The Biotin-PD-L1 aptamer, Cy3-PD-L1 aptamer, Scambled-PD-L1 aptamer were all synthesized by Sangon Biotech Co., Ltd. (Shanghai, China) The sequences are shown in Table S1. Streptavidin-ALP made by Bioss Antibodies (catalog number: bs-0437p-AP). L-Argnine, Sodium Chloride, and Magnesium chloride made by Macklin Biochemical Co., Ltd. (Shanghai, China) Tween-20 made by Beyotime. To rotate the tubes we used 3D Rotary made by MIULAB. Water purified by Mili-Q portable benchtop system was used throughout all the experiments and buffers preparation.

### 2.2. Cell Culturing for EVs Isolation

Human lung adenocarcinoma cells (A549) and Human lung epithelial cells (BEAS-2B) by Chinese Academy of Sciences were cultured (at 37 ${}^{\circ }$C in 5% CO${}_{2}$ and 95% air) in Dulbecco’s modified Eagle medium (DMEM, Gibco) medium augmented with 10% Fetal Bovine Serum (FBS, Gibco). After achieving the 70% confluency, cells were washed with PBS then DMEM with 10% exosomes depleted FBS (System Bioscience) was poured to support the growth of the cells. After 24 h to 48 h duration, the media was collected to amass EVs via differential ultracentrifugation [27]. In short, culture media was exposed to centrifugation (Beckman Coulter, Allegra X-30R) at 300× *g* for 10 min, then 2000× *g* for 10 min, and 10,000× *g* for 30 min to mitigate unwanted components such as cells, dead cells, and cell debris, respectively. Upon completion of the above steps, the supernatant was filtered via 0.2 µm syringe cap filter then the filtered supernatant was ultracentrifuged (100,000× *g*) for 90 min (Beckman Coulter, Optima XPN-100). After heedful pouring of supernatant the pellet was resuspended in 1 mL PBS and exposed to ultracentrifugation (100,000× *g*) for 90 min using TYPE 90 Ti Rotor (Beckman). Finally, supernatant was poured out and EVs’ pellet was re-suspended in 100 μL PBS (0.22 µm filtered) and stored at −80 ${}^{\circ }$C to be used later in further experiments. All the centrifugation and ultracentrifugation steps were performed at 4 ${}^{\circ }$C.

### 2.3. Fe${}_{3}$O${}_{4}$@TiO${}_{2}$ Beads Formation

Fe${}_{3}$O${}_{4}$@TiO${}_{2}$ beads were made following an established protocol [28,29]. The synthesis include the mixing of 0.05 g Fe${}_{3}$O${}_{4}$ with 0.3 mL aqueous Ammonia (28 wt%) in a 100 mL Ethanol solution. The amalgam was stirred (600 rpm) for 15 min at 45 ${}^{\circ }$C in a three neck flask. Then about 0.75 mL Tetrabutyl titanate solution made in Ethanol was introduced drop-wise into the solution under continuous stirring for almost 24 h at 45 ${}^{\circ }$C. Later in a Teflon-lined stainless-steel autoclave, the mixture was exposed to hydrothermal treatment at 180 ${}^{\circ }$C (24 h). The final product was then washed with water and Ethanol (10 mL) and dried overnight. After drying the final product was stored for subsequent experiments.

### 2.4. EVs Morphology, Characterization and Enumeration

Transmission electron microscopy (TEM) was used for visualization of EVs morphology. To accomplish this pipetted 20 μL EVs over a copper mesh and the superficial liquid was mitigated via filter paper. Then about 2% phosphotungstic acid negative staining solution was applied for 5 min. The copper mesh was then heedfully washed (5X) with double distilled water and dried prior to visualization via JEM—2200CX TEM (JEOL). Protein concentration of the samples was measured using Qubit fluorometer (Invitrogen) for westren blotting (WB). EVs and cells were lysed by heating for 5 min at 95 ${}^{\circ }$C in the loading buffer that were then loaded on 10% SDS-PAGE gel (Sangon Biotech) for separating the proteins. Polyvinylidene Difluoride (PVDF) membrane (Sangon Biotech) was selected for transferring the protein bands from gel to the membrane. After transferring, the membrane was blocked using 2% non-protein blocking solution for 2 h. The membrane was washed (3X) before dispersing in the solutions of primary antibodies (CD9, TSG101, Calnexin [ab275018 by Abcam] and PD-L1 antibody by Proteintech) and kept at 4 ${}^{\circ }$C for 12 h. After incubation with primary antibodies the membrane was then washed (3X) to remove unbound primary antibodies and incubated for 2 h with solution of secondary antibody (goat anti-rabbit, Abcam) at room temperature. Finally, enhanced chemiluminescence kit (Sangon Biotech) was used to visualize the protein bands. To get the information about the concentration and size distribution of EVs employed an NTA technique (ZetaView, Particle Metrix, Diessen, Germany). NTA readings for each sample were measured at 11 different positions. The result were visualized using ZetaView® software. All the frames were automatically analyzed to drop out outlier positions and calculated the EVs concentration and mean size.

### 2.5. Confocal Microscopy and Zeta Potential

For confocal microscopy EVs bound Fe${}_{3}$O${}_{4}$@TiO${}_{2}$ beads were blocked using blocking solution. The blocking solution was made by mixing 5% Bovine serum albumin (BSA, Sangon Biotech, Shanghai, China) and 0.5 mg/mL Salmon sperm DNA (Invitrogen) in HEPES buffer. After blocking added Cy3-PD-L1 aptamer and incubated for 30 min. The unbound aptamers were removed by washing (3X) and the beads + EVs + Cy3-PD-L1 aptamer were re-suspended in 300 μL PBS and examined using confocal microscope (Leica microsystems, 100X oil immersion objective). PBS was used as a control. For Zeta potential of Fe${}_{3}$O${}_{4}$@TiO${}_{2}$ beads (0.1 mg/mL), A549 released EVs ($10^{6}$ EVs/mL) and Fe${}_{3}$O${}_{4}$@TiO${}_{2}$+ EVs complex (0.1 mg/mL) used Zetasizer nanoseries (Nano-ZS).

### 2.6. EVs Accumulation by Fe${}_{3}$O${}_{4}$@TiO${}_{2}$ Beads

To measure the EVs enrichment capability of the Fe${}_{3}$O${}_{4}$@TiO${}_{2}$ beads, a PKH26 dye-stained A549 EVs were used [28]. PKH26 staining of A549 EVs was achieved following manufacture instructions. Samples containing equal concentration of PKH26-stained A549 EVs ($10^{10}$ EVs/ mL) were made and recorded the fluorescence. The stained EVs were then incubated with Fe${}_{3}$O${}_{4}$@TiO${}_{2}$ beads a magnetic rack was used to pellet Fe${}_{3}$O${}_{4}$@TiO${}_{2}$/EVs and measured the supernatant fluorescence. The experiment was conducted in triplicate and the capture efficiency was calculated as capture efficiency(%) = (F2/F1) × 100; where F1 is the fluorescence of EVs solution before enrichment by Fe${}_{3}$O${}_{4}$@TiO${}_{2}$ beads and F2 after enrichment by Fe${}_{3}$O${}_{4}$@TiO${}_{2}$ beads. Likewise, optimal quantity of Fe${}_{3}$O${}_{4}$@TiO${}_{2}$ beads and optimal time needed for achieving optimal capturing efficiency were also evaluated.

### 2.7. Clinical Feasibility

Clinical feasibility was elucidated using the serum samples of healthy individuals (n = 7) and LC patients (n = 18). After acquiring written consent and prior ethical approval from the ethical committee, we obtained serum samples at the Clinical laboratory of the Drum tower hospital affiliated with the Nanjing Medical University. The serum was filtered using 0.22 µm filter fitted as a syringe cap. About 50 µL of the undiluted serum was incubated with the Fe${}_{3}$O${}_{4}$@TiO${}_{2}$ beads for the duration of 10 min to capture EVs and followed the remaining steps of sensing assay to accomplish the detection. PBS was used as a blank control. For verification purpose all the samples were tested in triplicate.

### 2.8. Statistical Testing

To perform graphing and statistical operations used GraphPad prism software (Inc., La Jolla, CA, USA).

## 3. Results

### 3.1. Principal Design of the Sensing Assay

**Figure 1:**
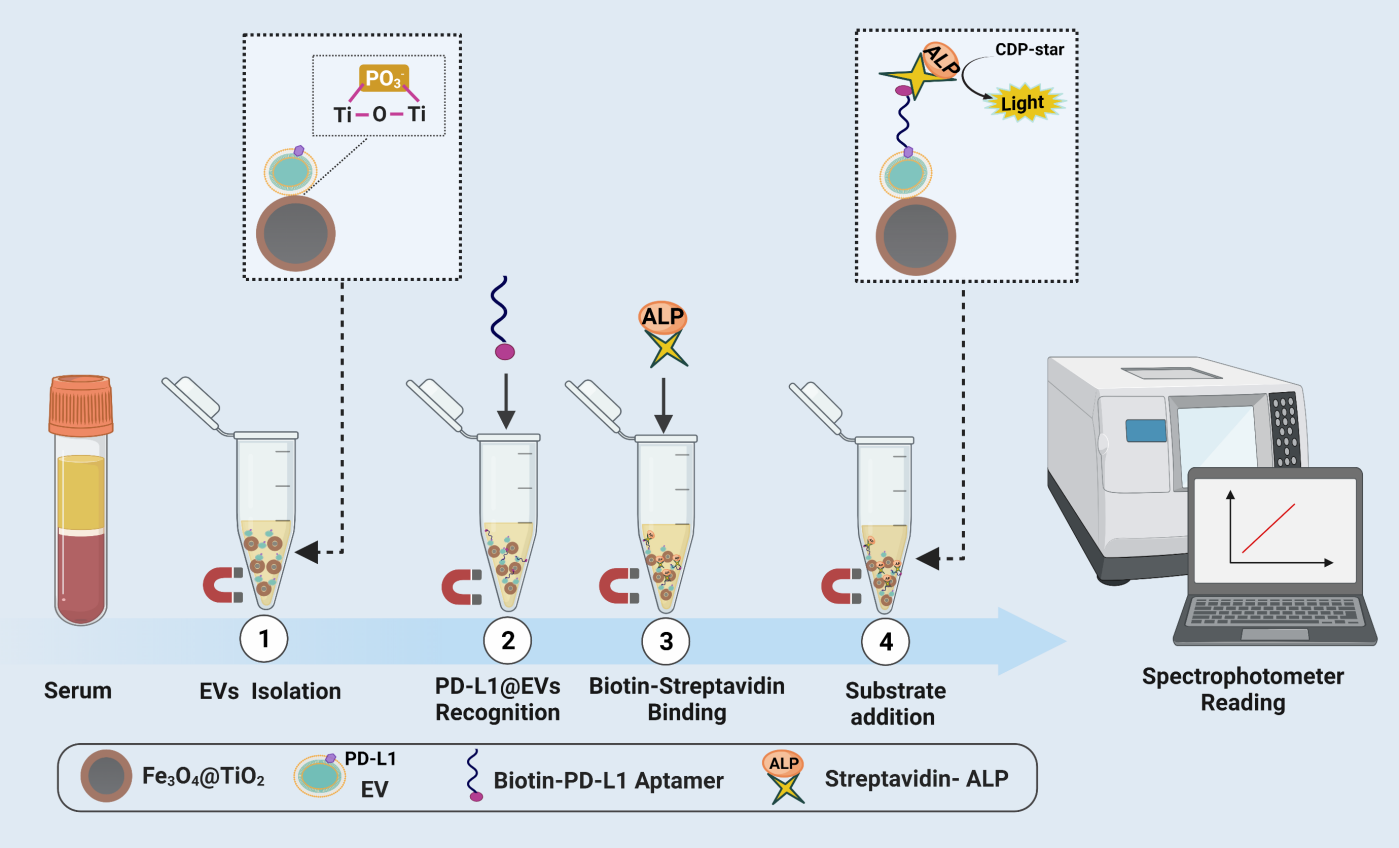
Principal design of the assay. (**1**) Fe${}_{3}$O${}_{4}$@TiO${}_{2}$ based enrichment of EVs from undiluted serum. (**2**) Addition of Biotin-PD-L1 Aptamer to bind to PD-L1@EVs. (**3**) Addition of Streptavidin-Alakaline phosphatase (ALP) to achieve Biotin-Streptavidin binding. (**4**) Addition of CDP-Star for catalysis by ALP and subsequent reading of chemiluminescense by spectrophotometer.

Figure 1 depicts the principal design of the sensing assay. First of all the undiluted serum was incubated with Fe${}_{3}$O${}_{4}$@TiO${}_{2}$ for 10 min to capture EVs. After capturing EVs the beads were pelleted using magnetic force to remove the supernatant. To block the non-specific binding sites a blocking solution containing salmon sperm DNA (0.5 mg/mL) and BSA (3% Vol/Vol) in HEPES buffer (10 mM) was added and incubated for 1 h with gentle rotation. The beads were then pelleted to remove the blocking solution and washed (TBS, pH 7.5 + 0.001% Tween + 1 mM MgCl${}_{2}$). The remaining sites were blocked by adding 500 mM Arginine in HEPES buffer for 30 min. After magnetic pelleting of beads and removal of supernatant Biotin-PD-L1 aptamer (0.075 µm in 3% BSA-HEPES, the binding buffer) was added and incubated for 30 min with gentle rotation. Magnetically pellet the beads to rinse the excessive aptamers followed by washing and addition of Streptavidin-ALP (1 mg/mL, dilution 1:3000) for about 20 min. Similarly, the beads were pelleted and the excessive Streptavidin-ALP was rinsed followed by the addition of CDP-Star: a chemiluminescent substrate of ALP and was gently rotated in the dark for 8–10 min. Finally, Fe${}_{3}$O${}_{4}$@TiO${}_{2}$ beads were magnetically separated and the supernatant was pipetted into the 96-wells half area white-plates. The chemiluminescence was recorded at 466 nm using a spectrophotometer (SpectraMax M5). The whole sensing assay completes at room temperature in less than 3 h.

### 3.2. Fe${}_{3}$O${}_{4}$@TiO${}_{2}$ Formation Analysis

Formation of the Fe${}_{3}$O${}_{4}$@TiO${}_{2}$ beads was analyzed using TEM (Figure S1A,B) and SEM (Figure S1C,D). The size of the beads was from 300 nm to 500 nm. The beads appear to have a black core of Fe${}_{3}$O${}_{4}$ and granulated surface coating of TiO${}_{2}$. Energy-dispersive X-ray spectroscopy based elemental analysis of Fe${}_{3}$O${}_{4}$@TiO${}_{2}$ beads confirmed the presence of Ti and Fe that also establish a successful coating of TiO${}_{2}$ over Fe${}_{3}$O${}_{4}$ beads (Figure S2). Fe${}_{3}$O${}_{4}$@TiO${}_{2}$ beads were then harnessed to capture EVs from PBS/serum based on TiO${}_{2}$ binding affinity with the phosphate group of the EV’s lipid bilayer [12]. The Zeta potential of the Fe${}_{3}$O${}_{4}$@TiO${}_{2}$ beads showed considerable change upon binding the negatively charged EVs. Hence, the changes in Zeta potential of Fe${}_{3}$O${}_{4}$@TiO${}_{2}$ beads can also transcribe the binding of EVs with beads (Figure S3).

### 3.3. EVs Characterization, Enumeration and PD-L1 Realization

**Figure 2:**
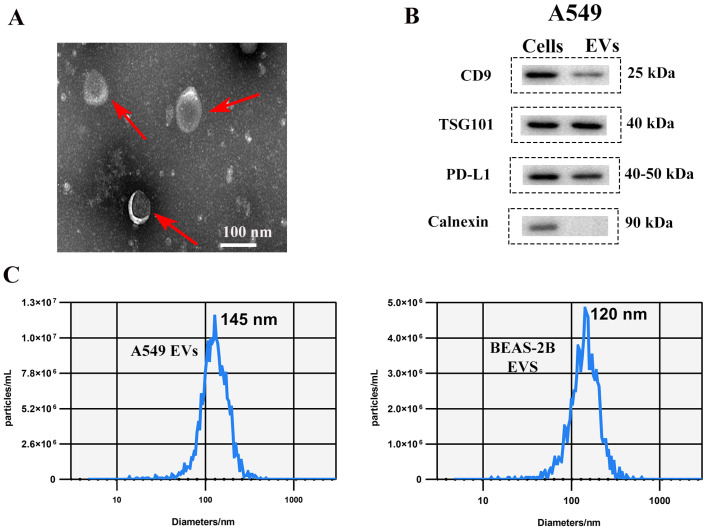
The characterization of canonical features of EVs. (**A**) TEM image of A549 released EVs red arrow points at EVs (**B**) western blot-based visualization of EVs’ positive and negative markers and PD-L1 marker. (**C**) NTA graph showing EVs concentration and size distribution.

The morphological characterization by TEM confirmed the typical saucer-shaped morphology of the acquired EVs ( Figure 2A). WB results validated the expression of EVs specific markers and cancer specific marker, we obtained distinct bands for CD9, TSG101 and PDL-1 verifying the presence of the EVs specific markers and cancer specific marker, respectively, while calnexin as an EVs’ negative marker (Figure 2B). NTA analysis showed that we had acquired around $2.5\times 10^{11}$ EVs/mL from the A549 cells with the mean size range of 145 nm. Likewise, from BEAS-2B cells acquired $4.5\times 10^{9}$ EVs/mL with 120 nm mean size (Figure 2C). NTA results verify the fact that cancer cell releases more EVs than normal cells.

### 3.4. Assay’s Conditions Optimization

**Figure 3:**
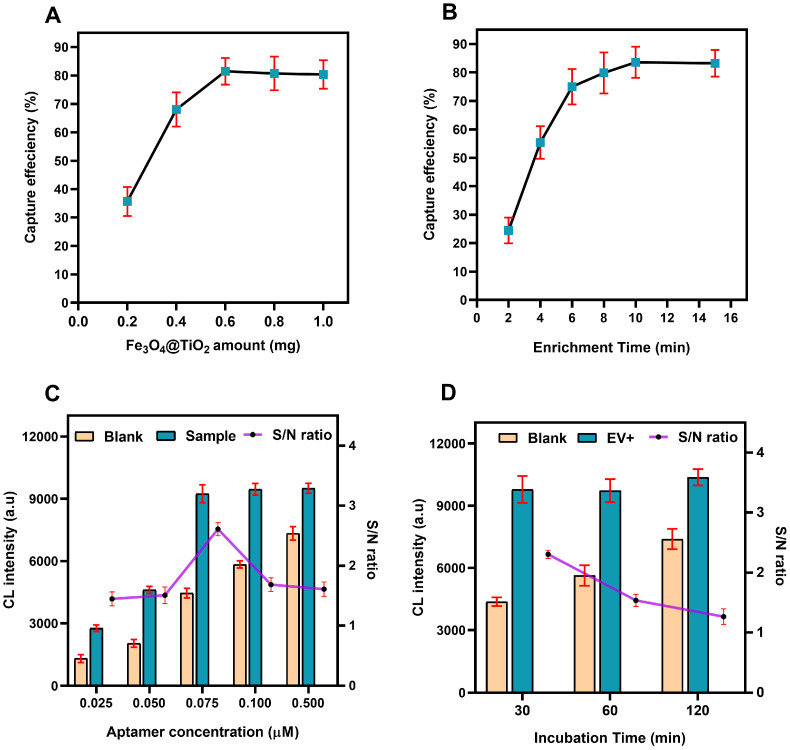
Optimization experiments. (**A**) Fe${}_{3}$O${}_{4}$@TiO${}_{2}$ amount and optimal capture efficiency. (**B**) Fe${}_{3}$O${}_{4}$@TiO${}_{2}$ incubation time and optimal capturing potential. (**C**) Aptamer concentrations for optimal performance of the assay. (**D**) Aptamer incubation time for optimal assay performance. (Error bar indicates the standard deviation of three replicates, S/N = signal-to-noise ratio).

In order to establish an excellent detection assay optimization of the key steps is crucial. One of the key step of the designed sensing assay is the capturing of EVs by Fe${}_{3}$O${}_{4}$@TiO${}_{2}$ beads. We therefore, optimized the amount of beads and incubation time to achieve optimum capture efficiency of Fe${}_{3}$O${}_{4}$@TiO${}_{2}$ beads for EVs. Different quantities of beads (0.2 mg, 0.4 mg, 0.6 mg, 0.8 mg and 1 mg) were tested to obtain the quantity of beads needed to achieve the highest capture efficiency for PKH26 labeled EVs. The florescence was recorded before and after the addition of Fe${}_{3}$O${}_{4}$@TiO${}_{2}$ beads, optimum capture efficiency (more than 80%) was obtained for 0.6 mg Fe${}_{3}$O${}_{4}$@TiO${}_{2}$ amount. Further increase in the amount showed no significant increment in capture efficiency, as shown in Figure 3A. Another important parameter was the optimization of incubation time needed for the Fe${}_{3}$O${}_{4}$@TiO${}_{2}$ beads to achieve maximum capturing using PKH26 labeled EVs, various time duration from 2 min to 16 min were tested for optimal capture efficiency. Maximum capture efficiency was recorded at 10 min and no significant increment was achieved in capture efficiency post 10 min (Figure 3B).

PD-L1 aptamer has a central role in the design of this study, therefore, it was pertinent to evaluate its optimum concentration for optimal assay performance. Different concentrations of aptamers(0.025, 0.050, 0.075, 0.100, 0.500 µM) were tested to find the optimum concentration of aptamers. The concentration of EVs ($10^{10}$ EVs/mL) was kept constant while PBS was used as blank. The best signal (sample CL intensity) to noise (PBS, CL intensity) was obtained for aptamer concentration of 0.075 µm as shown in Figure 3C. Similarly, to record best incubation time needed for the aptamer to produce good signal (CL intensity (a.u.) of EVs sample) as compared to the noise (CL intensity (a.u.) of the PBS). Three different incubation times (30 min, 60 min, 120 min) were trialed and best S/N ratio was recorded for 30 min incubation time. However, incubation times longer than 30 min resulted in increased background signals as shown in Figure 3D. To further study the specificity of the PD-L1 aptamer we compared it with scrambled PD-L1 aptamer and PBS (Figure S4). High CL intensity was obtained for PD-L1 aptamer as compared to scrambled aptamer and PBS. Validating PD-L1 aptamer high specificity.

### 3.5. Confocal Microscopy-Based Visualization of PDL@EVs

**Figure 4:**
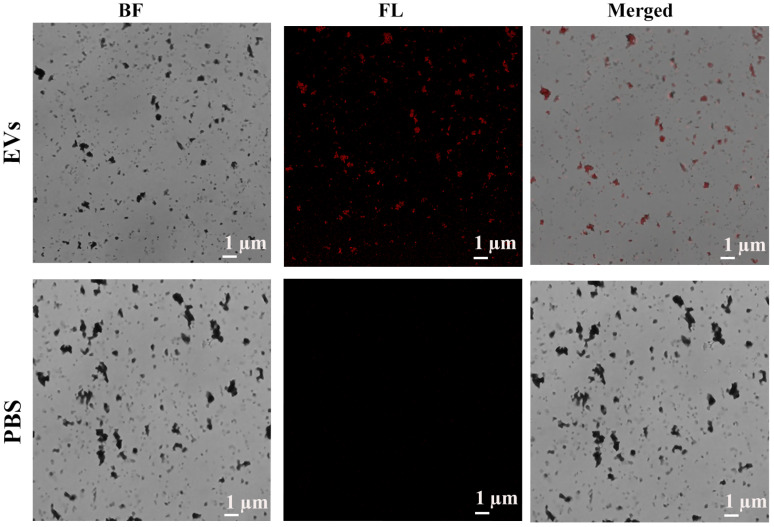
Confocal microscopy-based visualization of the PD-L1@EVs by Cy3-PD-L1 aptamer. BF (Bright filed), FL (Florescence).

To verify the presence PD-L1@EVs we used confocal microscopy. Cy3-PD-L1 aptamer was combined with two combinations; (1) Fe${}_{3}$O${}_{4}$@TiO${}_{2}$ with EVs and (2) Fe${}_{3}$O${}_{4}$@TiO${}_{2}$ with PBS. Images obtained showed Cy3 specific fluorescence in the first combination while no fluorescence can be seen in the image of the second combination. From the images we can rightly conclude the capturing of EVs by Fe${}_{3}$O${}_{4}$@TiO${}_{2}$ and can deduce the presence of PD-L1 over the surface of EVs as Cy3-PD-L1 aptamer can only bind to EVs expressing PD-L1 marker (Figure 4).

### 3.6. Verification of Assay’s Specificity, Sensitivity and Accuracy

**Figure 5:**
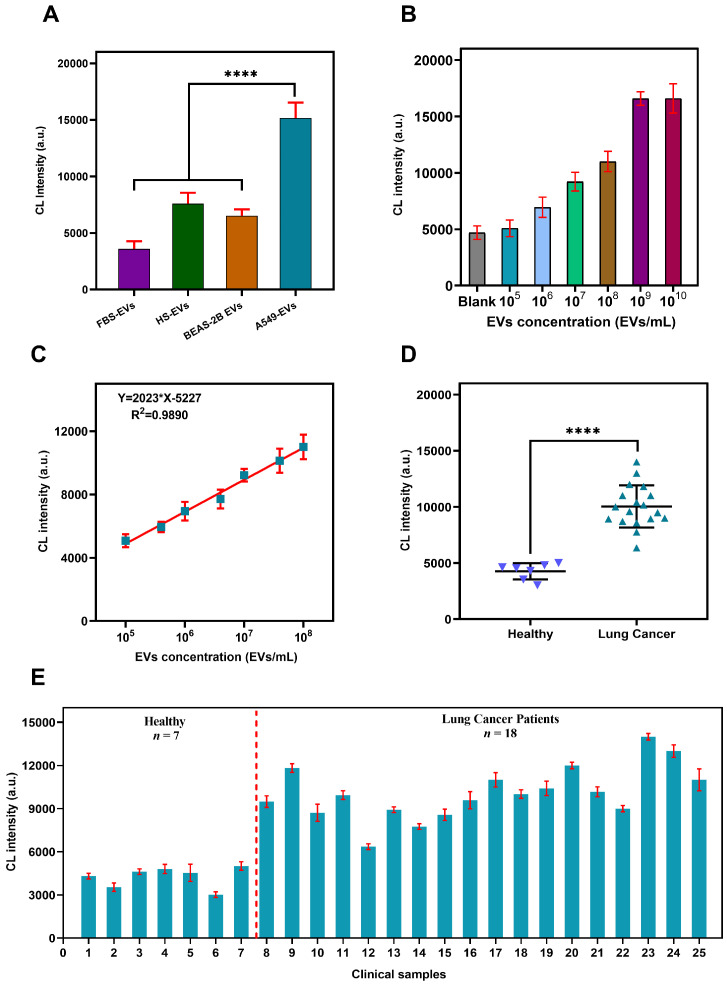
Assay features validation. (**A**) Assay specificity for PDL@EVs. HS (healthy serum) FBS (Fetal bovine serum). (**B**) Assay performance on different concentration of EVs. (**C**) Assay’s linear fitting between CL intensity and concentration of EVs. (**D**,**E**) Assay performance validation using clinical serum samples. (Error bar indicates standard deviation of three replicates, **** = *p* ≤ 0.0001).

Important features of an excellent assay are its specificity, sensitivity and accuracy. To test the specificity, the same concentration ($10^{8}$ EVs/mL) of cancer cell (A549) derived EVs and normal cells (BEAS-2B) derived EVs, FBS derived EVs, EVs derived from the serum of healthy individual were used. The results showed high CL intensity (a.u.) value for the A549 derived PD-L1@EVs as compared to the rest of the trailed EVs, establishing high specificity of sensing assay for PD-L1@EVs (Figure 5A).

Next, to assess the assay sensitivity different concentrations of the A549 released EVs were tested from $10^{5}$–$10^{10}$ EVs/mL. The results showed a direct relationship between the concentration of PD-L1@EVs and CL intensity (a.u.) value (Figure 5B). Increase in EVs concentration increase the CL intensity (a.u.). The calibration curve was drawn by plotting the values of CL intensity (a.u.) against the EVs concentration. Linear relationship existed between CL intensity and the EVs concentration from $10^{5}$–$10^{8}$ EVs/mL. The LOD of the method was calculated to be $2.85\times 10^{5}$ EVs/mL, using 3 times the standard deviation of the blank formula (Figure 5C).

To explicate the assay recovery ability, various concentrations of EVs ($10^{6}$, $10^{7}$ and $10^{8}$ EVs/mL) harvested from A549 cells were spiked into the EVs depleted healthy human serum. To calculate the percent (%) recovery; divided the detected EVs concentration by the concentration of EVs spiked into the serum multiplied by 100. It can be transpired from Table S2 that the assay has good recovery (≥90%) for all the tested samples. For any biological assay, its ability to produce consistently good results is of utmost importance. We trailed the assay using the same number of EVs ($10^{7}$ EVs/mL) consistently for 5 times. The results were clustered around the mean value, with the relative standard deviation of 4.41% (Figure S5).

### 3.7. Sensing PD-L1@EVs in Clinical Samples

An important feature of any detection method is its capability to perform well when tested using clinical serum samples; serum of healthy individuals (n = 7), and lung cancer patients (n = 18). The clinical information of the subjects used in the study are tabulated in Table S2. It is evident from Figure 5D,E that serum samples of healthy individuals have lower CL intensity value than lung cancer patients. The statistical significance of CL intensity (a.u.) value between two groups was analyzed using the Mann-Whitney U test (*p* < 0.001). These findings are in accordance with an already reported research that serum of LC patients have more PD-L1@EVs than the serum of healthy individuals [28]. To put it simple, the proposed assay is capable of differentiating between healthy and LC patients. Moreover, by taking into consideration the good performance of the sensing assay using clinical serum samples, it can be anticipated that the assay has good prospects for application in clinical setups for the detection of LC.

## 4. Discussion and Conclusions

Lung cancer (LC) rapid expansion in terms of new cases, its poor 5-year survival rate and towering mortality rate increased the demand for development of simple, sensitive, and non-invasive detection strategies [6]. EVs have been reported to be ideal for development of non-invasive diagnostic methods with excellent applicability [7]. PD-L1@EVs have been reported to be involved in immunosuppression and tumor immune escape [3]. Likewise, the quantity of PD-L1@EVs can be a biomarker for cancer and for evaluating the patients response to anti-PD-1/PD-L1 immunotherapy [5]. Taking into consideration the enormous potential of PD-L1@EVs as a biomarker [30], we designed a simple, specific and sensitive sensing assay for PD-L1@EVs. The salient features of the sensing assay are the Fe${}_{3}$O${}_{4}$@TiO${}_{2}$ beads remarkable capability to capture EVs from the undiluted serum (50 µL), hence, eliminates the use of ultracentrifugation and other commercial methods for EVs isolation prior to detection. The use of aptamer instead of antibodies as a recognition element, have added advantages such as aptamer have excellent sensitivity, specificity and affinity towards the target and are less expensive. The use of CDP-star as an ALP substrate eliminates the need for H${}_{2}$O${}_{2}$ as a co-substrate thus minimizing the interference and cost. The assay exhibited good sensitivity with an LOD $2.54\times 10^{5}$ EVs/mL and a wider linear range ($10^{5}$–$10^{8}$ EVs/mL). The performance of the assay remained significant in complex undiluted clinical serum samples establishing its utility for clinical samples. The sensing assay completes at room temperature without any expensive machine-based pretreatment.

The LOD and linear range of the sensing assay is better than rest of tabulated methods in Table 1. Most of the methods employed ultracentrifugation or other commercial kit prior to detection of EVs in clinical serum, some used simulated serum, and some used diluted serum with spiked EVs or just detected using cells culture medium. However, the proposed sensing assay showed good performance with real undiluted serum samples. Compared to the traditional ELISA assay for PD-L1@EVs reported before our sensing assay needs lesser time (less than 3 h). They have used costly EVs isolation kit, and the operation time is over 12 h [3]. Another method based on using the digital droplet PCR and aptamer for the detection of PD-L1@EVs had achieved good sensitivity. However the method need large sample volume and employed ultracentrifugation to enrich EVs prior to detection. The process of ultracentrifugation need an expensive instrument and is time consuming [3]. Zhang et al., achieved lower LOD but narrow liner range compared to the proposed sensing assay. They have also employed ultracentrifugation prior to detection of EVs that lengthens the detection operation [31]. Recently published research establish that PD-L1@EVs can be used for the diagnosis of LC [28] and breast cancer [32]. Our results also recognize the fact that the amount of PD-L1@EVs is distinct between healthy individuals and LC patients. PD-L1 has been reported to undergo heavy glycosylation blocking the binding of antibody; affecting the performance of antibody based analytical methods for PD-L1@EVs detection [33]. However, aptamers due to small size can overcome the PD-L1 glycosylation interference and can bind efficiently [5]. Thus, the use of aptamer in our study increases the sensitivity as well as decreases the cost compared to the antibody-based methods. To sum it up, we have developed a simple Fe${}_{3}$O${}_{4}$@TiO${}_{2}$ and aptamer-based chemiluminescent sensing assay for EVs with PD-L1 marker for LC diagnosis. To conclude we have developed a simple, specific and sensitive chemiluminsent sensing assay for PD-L1@EVs by integrating Fe${}_{3}$O${}_{4}$@TiO${}_{2}$ beads rapid enrichment of EVs from undiluted serum with PD-L1 aptamer specificity and cheminluminsence sensitivity. The assay has good prospects for application in clinical setups to classify between healthy and LC patients; it can also aide in the surveillance of patients’ response to anti-PD-1/PD-L1 immunotherapy. In future we are looking to automate the assay for improving the sensitivity and linear range and to eliminate the loss of EVs due to differences in inter-personal pippeting skills during washing steps. In addition by using a large patients cohort data integrated with machine learning model for results analysis is recommended to further validate and improve the utility of our method for LC diagnosis. In order to expand the assay utility for surveillance of anti-PD-L1/PD-1 immunotherapy we are planning to collaborate with hospital to test the sensing assay using clinical serum samples of LC patients undergoing anti-PD-L1/PD-1 immunotherapy.

## Figures and Tables

**Table 1:** Comparison with contemporary methods.

Method	LOD (EVs/mL)	Sample Type	Prior Isolation (UC/kit/UF)	Linear Range (EVs/mL)	Reference
Fluorescence	$1.6\times 10^{6}$	EVs Spiked Serum	Yes	$1.66\times 10^{6}$–$1.6\times 10^{8}$	[34]
Fluorescence	$7.6\times 10^{6}$	Culture Medium	Yes	$1.68\times 10^{7}$–$4.2\times 10^{10}$	[35]
FET	$1.76\times 10^{8}$	Diluted Serum	Yes	$1.76\times 10^{8}$–$1.76\times 10^{10}$	[36]
Optical	$10^{8}$	EVs Spiked Serum	Yes	$3.9\times 10^{7}$–$2\times 10^{10}$	[37]
Lateral flow immunoassay	$8.54\times 10^{8}$	Culture Medium	Yes	$10^{9}$–$10^{11}$	[38]
Colorimetric	$5.2\times 10^{8}$	Diluted Serum	Yes	$1.84\times 10^{9}$–$2.2\times 10^{10}$	[39]
Colorimetric	$13.52\times 10^{8}$	Diluted Serum	Yes	$1.9\times 10^{9}$–$3.38\times 10^{10}$	[14]
Chemiluminescence	$2.63\times 10^{8}$	Undiluted Serum	No	$2.92\times 10^{8}$–$2.80\times 10^{11}$	[26]
Chemiluminescence	$2.85\times 10^{5}$	Undiluted Serum	No	$10^{5}-10^{8}$	**This method**

## Data Availability

Data supporting the research findings are provided in the main manuscript and supporting information.

## Supplementary Materials

The following supporting information can be downloaded at: https://www.mdpi.com/article/10.3390/bios12050281/s1, Table S1. The aptamer sequences used in the sensing assay; Table S2. The recovery (%) of the assay; Table S3. Clinical information of the patients enrolled in the study; Figure S1. TEM and SEM images of Fe${}_{3}$O${}_{4}$@TiO${}_{2}$ beads; Figure S2. XDS analysis of Fe${}_{3}$O${}_{4}$@TiO${}_{2}$ beads; Figure S3. Zeta potential of Fe${}_{3}$O${}_{4}$@TiO${}_{2}$ beads, EV and Fe${}_{3}$O${}_{4}$@TiO${}_{2}$ beads with EVs. Figure S4. PD-L1 aptamer specificity; Figure S5. Repetitions for the confirming the analytical performance of the sensing assay.
