# Supplementary material for: Rapid Capturing and Chemiluminescent Sensing of Programmed Death Ligand-1 Expressing Extracellular Vesicles
Source: Biosensors (Basel). 2022 Apr 28;12(5):281. doi: 10.3390/bios12050281 (PMC9138745; doi:10.3390/bios12050281)
Supplement: Supplementary file 1 [file biosensors-12-00281-s001.zip › biosensors-1662131-supplementary.pdf]

Table S1. The aptamer sequences used in the sensing assay.

Name	Sequences (5' to 3')
**PD-L1**	AAGACGGACCAGCCTTGCCGCAAGACGGACCAGGGATT
**Biotin-PD-L1**	Biotin-AAGACGGACCAGCCTTGCCGCAAGACGGACCAGGGATT
**Cy3-PD-L1**	Cy3-AAGACGGACCAGCCTTGCCGCAAGACGGACCAGGGATT
**Biotin-PD-L1-srambled**	Biotin-ACGACATACCGGCCTTTCCGCAAGACGGACCAGGGGT

Table S2. The recovery (%) of the sensing assay.

Spiked (EVs/mL)	Recovery (%)
10^6	96.49 ± 3.59
10^7	95.85 ± 2.94
10^8	90.05 ± 3.14

Note: The readings were obtained in triplicate for each sample.

The data is shown as mean ± standard deviation (%).

Table S3. Clinical information of the patients enrolled in the study.

S/No	Age	Sex	CEA (ng/mL）	CA-125 （U/mL）	CYFRA21-1 (ng/mL）	NSE (ng/mL）	EGFR	T	N	M	Stage
1	45	M	N/A	N/A	N/A	N/A	N/A	N/A	N/A	N/A	N/A
2	56	F	N/A	N/A	N/A	N/A	N/A	N/A	N/A	N/A	N/A
3	37	M	N/A	N/A	N/A	N/A	N/A	N/A	N/A	N/A	N/A
4	43	M	N/A	N/A	N/A	N/A	N/A	N/A	N/A	N/A	N/A
5	42	M	N/A	N/A	N/A	N/A	N/A	N/A	N/A	N/A	N/A
6	39	M	N/A	N/A	N/A	N/A	N/A	N/A	N/A	N/A	N/A
7	57	F	N/A	N/A	N/A	N/A	N/A	N/A	N/A	N/A	N/A
8	66	M	1.06	21.7	2.66	18.5	+	T2b	N0	M0	IIB
9	65	M	2.07	23.7	3.89	16.9	+	T1b	N0	M0	IA2
10	63	M	3.53	28.2	2.64	15.9	+	T4	N1	M0	IIA
11	56	F	1.62	24.5	11.4	41.8	+	T2a	N2	M0	IIB
12	70	M	1.07	53.7	2.4	14.9	+	T1c	N0	M0	IA3
13	71	F	1.07	53.7	2.4	14.9	+	T1c	N0	M0	IA3
14	72	F	1.3	17.80	1.90	11.75	+	T2a	N0	M0	IB
15	56	F	0.97	15.4	1.03	12.2	+	T2a	N1	M0	IIB
16	64	M	3.53	28.2	2.64	15.9	+	T4	N1	M0	IIB
17	46	F	0.68	6.5	1.58	13.6	+	T2a	N0	M1	IV
18	66	M	0.58	28.3	3	11.7	+	T2b	N1	M1	IV
19	61	M	50.84	80	21.1	57.7	+	T3	N2	M1	IV
20	69	M	31.97	82.3	10.2	13.6	+	T2	N3	M1	IV
21	59	F	1.8	6.8	5.4	9.80	+	T3	N3a	M1	IV
22	57	M	7.07	35	3.84	9.88	+	T2	N3	M1	IV
23	61	M	50.84	80	21.1	57.7	+	T2	N1	M1	IV
24	61	F	43.19	89.4	2.71	12.8	+	T1	N3	M1	IV
25	73	M	>100	76	4.89	15.7	+	T4	N3	M1	IV

Note: *Carcinoembryonic antigen (CEA), Cancer antigen (CA)-125. cytokeratin 19 fragment antigen (CYFRA 21-1), Neuron-specific enolase (NSE), Epidermal growth factor receptor (EGFR), Tumor Size (T), Nodules (N), Metastasis(M), Not available (N/A).*

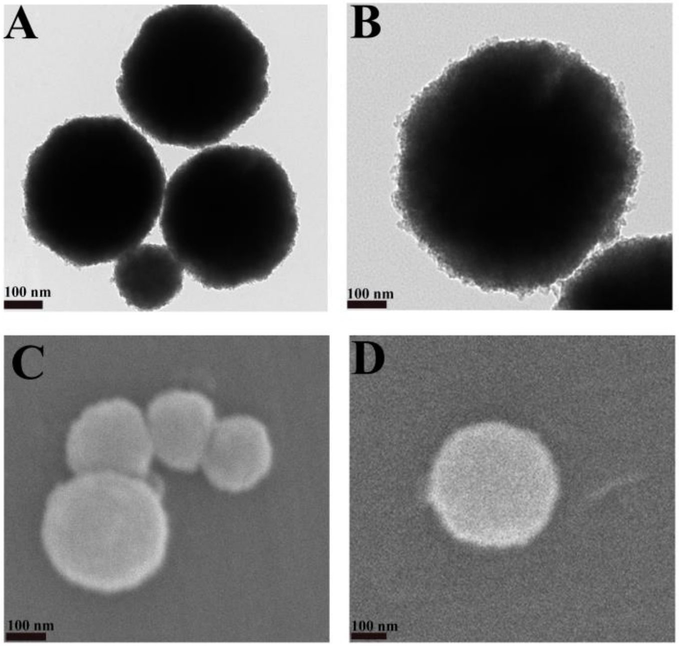

Figure S1. TEM and SEM images of $Fe_3O_4@TiO_2$. (A) multiple $Fe_3O_4@TiO_2$ (B) single $Fe_3O_4@TiO_2$ (C) multiple $Fe_3O_4@TiO_2$ (D) Single $Fe_3O_4@TiO_2$.

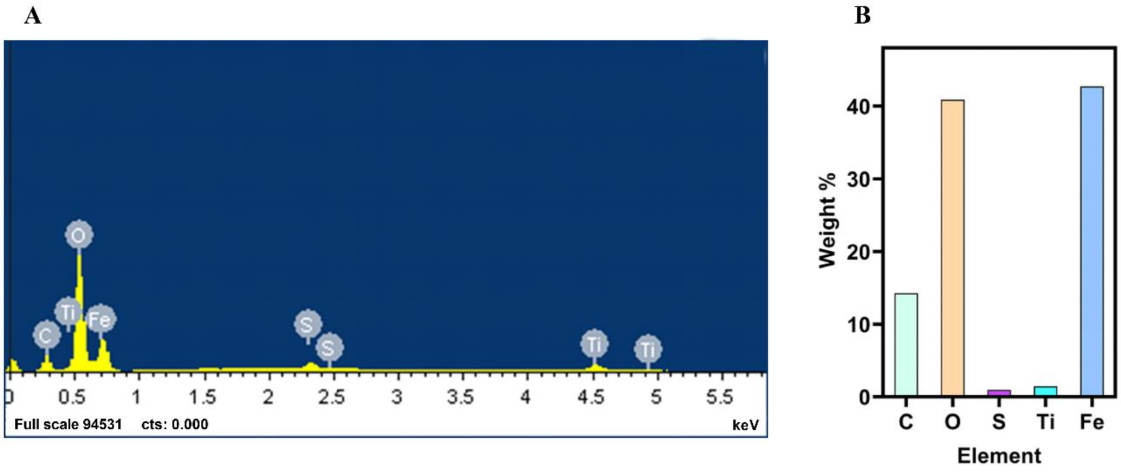

Figure S2. XDS analysis of $Fe_3O_4@TiO_2$.(A) XDS spectrum of the $Fe_3O_4@TiO_2$ beads (B) Percentage weight of respective elements.

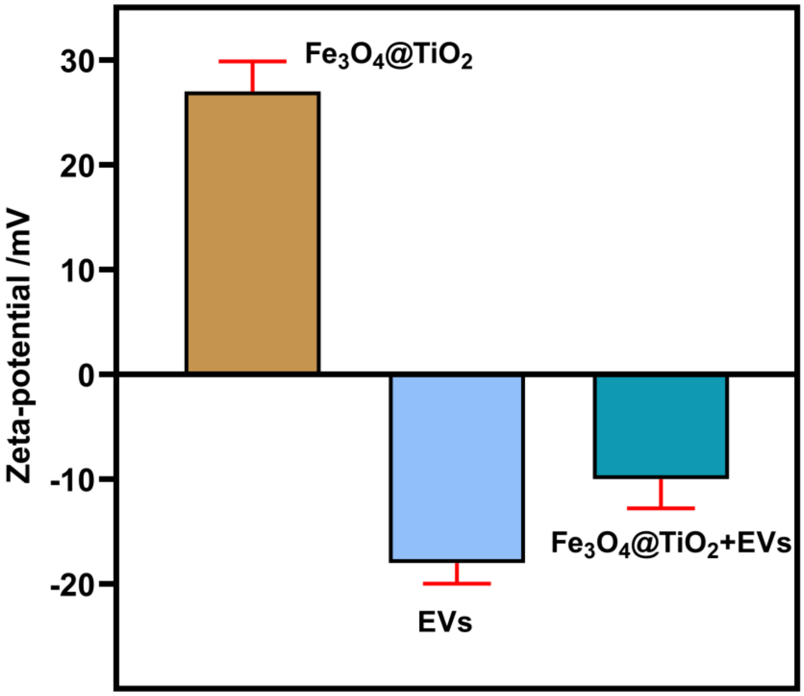

Figure S3. Zetapotential of $Fe_3O_4@TiO_2$ beads, EVs and $Fe_3O_4@TiO_2$ with EVs.

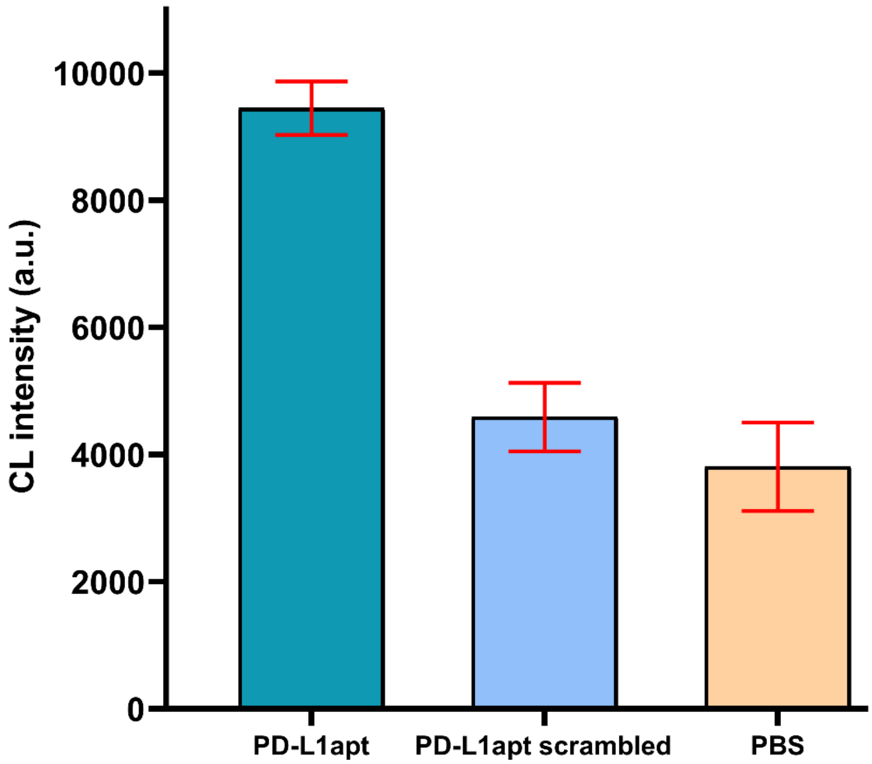

Figure S4. PD-L1 aptamer specificity.

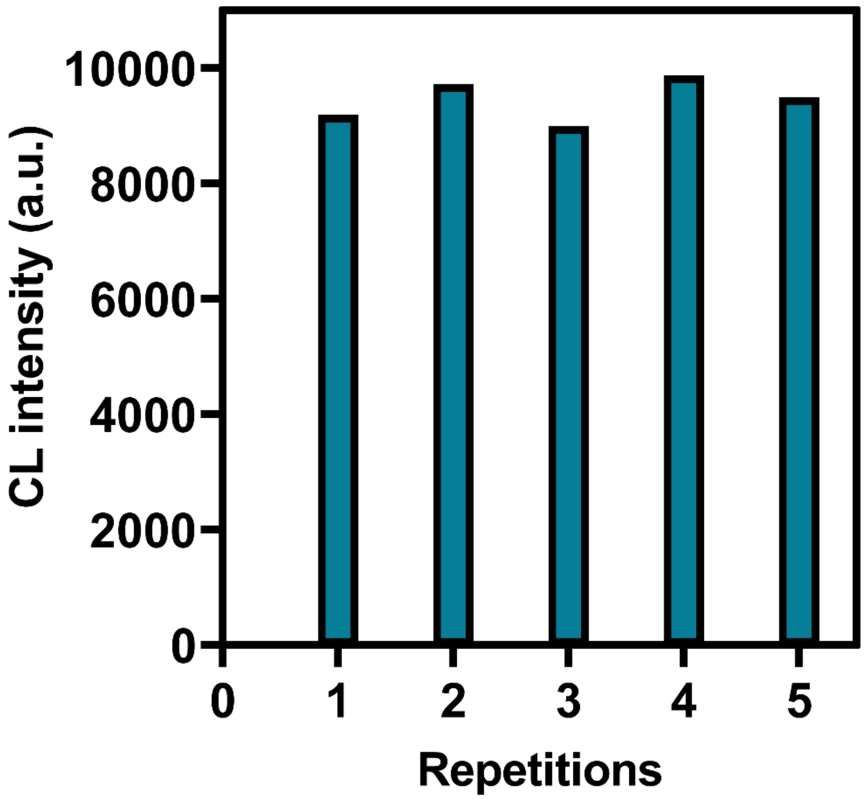

Figure S5. Repetitions for the confirming the analytical performance of the method. Repetitions (1-5) of 10^7 EVs/mL. (Relative standard deviation=**4.41 %,** mean value=9276.4).
